# THE ROUTINE USE OF THE METHYLENE BLUE TEST IN SLEEVE GASTRECTOMY: WHY NOT?

**DOI:** 10.1590/0102-672020210002e1612

**Published:** 2021-12-17

**Authors:** Álvaro A. B. FERRAZ, Fernando SANTA-CRUZ, João Victor BELFORT, Vladimir C. T. SÁ, Luciana T. SIQUEIRA, José Guido C. ARAÚJO-JÚNIOR

**Affiliations:** 1 Department of Surgery, Hospital de Clínicas, Federal University of Pernambuco, Recife, PE, Brazil; 2 Gastrointestinal Surgery Service, Esperança Hospital, Rede D'Or São Luiz, Recife, PE, Brazil; 3 Medical School, Federal University of Pernambuco, Recife, PE, Brazil; 4 General Surgery Service, Hospital Agamenon Magalhães, Recife, PE, Brazil

**Keywords:** Bariatric surgery, Fistula, Methylene blue, Cirurgia bariátrica, Fístula, Azul de metileno

## Abstract

**Background:**

Although considered a safe procedure, sleeve gastrectomy (SG) has a non-negligible risk of major postoperative complications related to it, with special attention to gastric leaks**.**

**Aim:**

Evaluate the clinical value of the methylene blue test (MBT) in predicting the occurrence of post-SG leaks.

**Methods:**

Retrospective study that included 1136 patients who underwent SG with intraoperative MBT between 2012 and 2016. Sensitivity, specificity, positive predictive value (PPV) and negative predicted value (NPV) were calculated to determine the clinical correlation between the MBT and the occurrence of postoperative leaks. Staple line oversewing was performed in all patients who presented positive MBT.

**Results:**

Laparoscopic SG was performed in 97.0% of cases; open in 2.3%, and robotic in 0.7%. MBT was positive in 19 cases (1.67%). One positive MBT occurred during an open SG and the other 18 at laparoscopy. Moreover, there were nine cases (0.8%) of postoperative leaks, among which, only two presented positive MBT. MBT diagnostic value was evaluated through the calculation of sensitivity (22.0%), specificity (98.0%), PPV (11.0%) and NPV (99.0%). There were no cases of allergic reaction or any other side effect with the use of the methylene blue solution.

**Conclusion:**

MBT showed high specificity and negative predictive value, thus presenting an important value to rule out the occurrence of postoperative leaks.

## RESUMO

**Racional:**

Embora considerada procedimento seguro, a gastrectomia vertical (GV) apresenta risco não desprezível de complicações pós-operatórias importantes relacionadas a ela, com atenção especial para fístulas gástricas

**Objetivo:**

Avaliar a aplicabilidade clínica do teste do azul de metileno (TAM) na predição da ocorrência de fístulas após a GV.

**Método:**

Estudo retrospectivo que incluiu 1136 pacientes operados entre 2012 e 2016 com aplicação do TAM intraoperatório. Sensibilidade, especificidade, valor preditivo positivo (VPP) e valor preditivo negativo (VPN) foram calculados na tentativa de determinar a correlação clínica entre os resultados do TAM e a ocorrência de fístulas pós-operatórias. Sobressutura da linha de grampos foi realizada em todos os pacientes que apresentaram TAM positivo.

**Resultados:**

GV laparoscópica foi realizada em 97,0% dos casos; por laparotomia em 2,3% e roboticamente em 0,7%. TAM foi positivo em 19 casos (1,67%). Um dos casos positivos ocorreu na laparotomia e os outros 18 na laparoscopia. Ainda, houve nove casos (0,8%) de fístulas pós-operatórias, dentre os quais, apenas dois apresentaram TAM positivo. O valor diagnóstico do TAM foi avaliado através dos cálculos de sensibilidade (22,0%), especificidade (98,0%), VPP (11,0%) e VPN (99,0%). Não houve casos de reação alérgica ou qualquer outro efeito colateral advindo do uso da solução de azul de metileno.

**Conclusão:**

TAM mostrou alta especificidade e VPN, apresentando importância em descartar a ocorrência de fístulas pós-operatórias.

## INTRODUCTION

Currently, sleeve gastrectomy (SG) is one of the most popular bariatric procedures worldwide and the most performed in the USA and Europe^4^ ^,^ ^17^. This is justified by its technical simplicity combined with the attracting results regarding weight loss and comorbidities resolution within the short and long terms^17^ ^,^ ^21^ ^,^ ^24^.

Although considered a safe procedure, there is a non-negligible risk of major postoperative complications related to this surgery, with special attention to gastric leaks, which are one of the most dreadful events, leading to postoperative morbidities, psychological distress and long hospital stay^1^ ^,^ ^15^ ^,^ ^25^. Leak rates after SG are varied, with reports from 0.9% to 7.0%^1^ ^-^ ^5^. In this context, it is highly encouraged that surgeons make use of safe approaches to avoid at maximum the occurrence of these events.

Gastric leaks of early onset usually occur due to stapler misfiring, direct tissue injury (secondary to surgeons' lack of expertise and learning curves) and/or patients' risk factors (including diabetes, smoking and vasculopathies), and, occasionally, these leaks can be caught intraoperatively and promptly closed^1^ ^,^ ^6^. In order to assess the staple line integrity during the procedure, several tests, such as the methylene blue test (MBT), have been used; however, showing conflicting results^1^ ^,^ ^13^ ^,^ ^18^. Although widely used, the evidence supporting the routine use of this test is scarce, and no study have yet confirmed its value in preventing post-SG leaks^18^.

Taking this scenario into consideration, the present study seeks to evaluate the diagnostic value of the intraoperative MBT in predicting staple line leaks following SG. We hypothesized that the MBT would be of useful in predicting the occurrence or not of post-SG leaks.

## METHOD

This research was approved by Institutional Ethics Committee - CAAE 29526320.9.0000. It is retrospective cohort included 1136 patients who underwent open, laparoscopic or robotic SG in our institutions between 2012 and 2016. All patients were operated by the same surgeon (AABF). This study included patients from both genders, aged between 18 and 65 years, who underwent SG with intraoperative MBT. Data was prospectively gathered from database.

Data obtained consisted of patients' demographics, including gender, age and preoperative BMI, besides the occurrence of intraoperative and postoperative leaks. Intraoperative leaks were defined as a positive MBT, namely, when the methylene blue solution was observed over the staple line. A negative MBT consisted of the non-observation of the methylene blue solution over the staple line. The primary endpoint of the current study was to assess the clinical value of the MBT in predicting postoperative leaks after SG.

After finishing the stapling of the stomach (calibrated over a 36 Fr bougie), a reinforcement through-and-through suture was performed all over the staple line with 3-0 Stratafix^TM^. Subsequently, methylene blue solution (1:10) was flushed through a 20 ml syringe into the remaining stomach through the previously inserted gastric bougie. In those cases where there was no evidence of methylene blue leaking over the staple line (MBT negative), an omentopexy was performed in the distal 2/3 of the remaining stomach and the surgery was completed. However, when the MBT was positive, the leaking site was oversewn with 3-0 Stratafix^TM^and a cavitary drain was placed before proceeding with the omentopexy. Drains were removed at the 10^th^postoperative day.

### Statistical analysis

A spreadsheet in Microsoft Excel was created to analyze the data, which was moved to a SPSS software, version 18, to perform the analysis. Next, percentage frequencies of the variables were calculated and the frequency distributions determined to evaluate the demographic profile of the patients. Results of the MBT and the occurrence of postoperative leaks were also analyzed through descriptive statistics. The diagnostic value of the MBT was determined through the calculation of sensitivity, specificity, positive predictive value (PPV) and negative predictive value (NPV).

## RESULTS

Overall 1136 patients who underwent SG, and to whom the MBT was applied, were analyzed. The sample consisted of 79.8% of women and 20.2% of men with mean age of 39.6±12.8 years. Mean preoperative BMI was 38.9±8.1 kg/m^2^. Type 2 diabetes was present in 458 (40.3%) patients and hypertension in 321 (28.2%). Laparoscopic SG was the most performed procedure, accounting for 1102 cases (97.0%). Open SG was performed in only 26 (2.3%), while robotic was performed in the remaining eight cases (0.7%). Patients' demographic profile is presented inTable 1. There were no cases of allergic reactions with the use of the MBT in our sample.

**TABLE 1:** Demographic data of the 1136 included patients

Patient characteristics	n=1136
Age (year)	39.6±12.8
Female gender, n (%)	907 (79.8)
BMI (kg/m^2^)	38.9±8.1
T2D, n (%)	458 (40.3)
Hypertension, n (%)	321 (28.2)
Laparoscopic SG n (%)	1102 (97.0%)
Open SG n (%)	26 (2.3%)
Robotic SG n (%)	8 (0.7%)

BMI=body mass index; T2D=type 2 diabetes; SG=sleeve gastrectomy

The MBT was positive in 19 cases (1.67%). One positive MBT occurred during an open SG and the other 18 at laparoscopic SG. Moreover, there were nine cases (0.8%) of postoperative leaks, among which, two had positive MBT, and seven negatives (Table 2). Also, was identified two types of intraoperative leaks (positive MBT): leaks around the reinforcement punctures (n=16) and jet leaks (n=3)^20^.

**TABLE 2:** Correlation between the results of MBT and occurrence of post-SG leaks

		Postoperative leak	
		Yes	No
MBT	Positive	2	17
	Negative	7	1110

MBT=methylene blue test

The assessment of MBT diagnostic value revealed that this test present high specificity and high NPV (98% and 99%, respectively) in predicting postoperative leaks. The analysis showed poor sensitivity and low PPV (22% and 11%, respectively); however, this could have been influenced by the fact that all positive cases were promptly submitted to staple line oversewn (Table 3).

**TABLE 3:** Diagnostic value of the MBT in predicting postoperative leaks following SG

Test properties	MBT (%)
Sensitivity	22
Specificity	98
PPV	11
NPV	99

MBT=methylene blue test; PPV=positive predictive value; NPV=negative predictive value; SG=sleeve gastrectomy

## DISCUSSION

SG is a safe and effective procedure; however, it is not free of complications, and leaks constitute a nightmare in the daily practice of the bariatric surgeon when performing this procedure. The theoretical benefit of intraoperative leak testing, such as the MBT, is that it allows the surgeon to oversew the staple line if an intraoperative leak is detected. However, until now, there is no proven correlation between oversewing and prevention of postoperative leaks^6^.

Wahby et al^26^ found, in their retrospective analysis, that the MBT helped to reduce the incidence of post-SG leaks from 3.93% to 1.4% by allowing the intraoperative repair of the leaking sites. Other studies have claimed that there is no advantage in performing the MBT during SG. Kirby et al^18^ failed in trying to establish a clinical value for the MBT in bariatric procedures. With a sample of 225 patients who underwent SG, they found three cases of postoperative leaks, all of which had negative MBT. Furthermore, Sethi et al^22^ found, also in a retrospective study, that among their 1329 cases who underwent SG, none presented positive MBT and, still, 15 patients developed postoperative leaks. Due to their results, both studies have discouraged the use of MBT.

It is important to remember that all kinds of intraoperative leaking tests have potential to identify intraoperative leaks, which generally occur due to stapler device misfiring or flawed surgical technique^6^. However, leaks, especially those of late onset (>5 days postoperatively), are a very complex entity that can develop from lots of other conditions (ischemic causes) and not only from an intraoperative leak that persisted^1^ ^,^ ^6^. Therefore, if a patient presents a negative MBT or any other intraoperative leaking test, it does not imply that this patient is "immune" from postoperative leaks. However, for those intraoperative leaks, the MBT helps the surgeon to identify flaws in the staple line and, subsequently, repair it.

In the present study, we found that the MBT presents high specificity (98.0%) and high NPV (99.0%), what confers to this test a high capacity to rule out the occurrence of persistent leaks in the postoperative period. Moreover, our results also showed a poor sensitivity (22.0%) and low PPV (11.0%), namely, there was no correlation between a positive MBT and the occurrence of a postoperative leak according to our results. However, we should emphasize that since all cases with positive MBT were submitted to staple line oversewing, these results regarding sensitivity and PPV may not be reliable without a control group to compare them.

Results regarding techniques of staple line reinforcement are still conflicting in the literature. Some have found that this strategy may reduce bleeding and leak rates^23^ ^,^ ^19^, while others have shown no benefits with it^11^ ^,^ ^16^. Analysis of the Metabolic and Bariatric Surgery Accreditation and Quality Improvement Program (MBSAQIP) data for 2012-2014 showed that staple line reinforcement techniques were associated with increased leak rates. However, the overall leak rate in the group of patients that underwent staple line oversewing isolated - without other reinforcement methods - was lower than in the group where no reinforcements were performed - neither staple line oversewing nor buttress material (0.59%x0.65%)^9^.

On the other hand, data from the MBSAQIP 2015-2016 Participant User File did not show any significant difference between the incidences of post-SG leaks with or without the use of staple line reinforcements^12^ ^,^ ^14^. Nevertheless, despite not being statistically significant, the overall leak rate after SG with staple line oversewing isolated was slightly lower than that reported after the procedure without any reinforcement techniques (0.3%x0.4%)^14^.

Rogula et al^20^ compared different reinforcement techniques in an in vitro study and found that the mean intragastric bursting pressure are higher in those cases where reinforcement sutures were performed; however, they showed that continuous Lembert's suture presented better results regarding leak rates when compared to through-and-through sutures. The rationale behind these findings is that through-and-through sutures create micro-perforations with the needle passage and may facilitate further leaks. However, in our sample, all of the 1136 patients were submitted to staple line reinforcement with a through-and-through suture line and the incidence of postoperative leak was still below 1%.

Furthermore, authors have also claimed that another reason not to perform the MBT is the risk of anaphylaxis^18^. Although a few cases of allergic reactions have been reported with the use of the methylene blue, there is no report of anaphylactic shock following the use of this dye^2^ ^,^ ^10^. In the present study, the MBT was performed in all patients and there were no cases of allergy-like reactions nor any other side effect. Therefore, it is reasonable to raise the hypothesis that the routine use of the MBT might be a safe practice.

Using the MBT was found an overall leak rate of 0.8% following SG, what is an acceptable rate (<1.0%) according with recent reports^3^ ^,^ ^8^. It is difficult to establish a trustworthy correlation between the MBT results and the occurrence of postoperative leaks, since if an intraoperative leak is detected by this test, measures are promptly taken in order to repair the defect. Through this perspective, it is possible that, if these defects were not repaired, persistent leaks would develop in the postoperative period. Therefore, we postulate that the MBT may have avoided a considerable part of those intraoperative leaks from persisting, and that those cases that presented negative MBT may have developed leaks for a reason other than stapling misfiring or flawed technique, including patients' risk factors.

There are some limitations implicated in the present study. The first is related to its retrospective nature, thus being prone to all biases of such studies. Another limitation is that we do not have a control group of patients who underwent SG without MBT and staple line oversewing. On the other hand, there are some strengths, such as the fact that all patients were operated by the same surgeon, what contributes to eliminate the possibility of technical biases related to learning curves and different surgeons' expertise. Another strong point is the size of our sample, comprised of 1136 patients, what reinforces the value of our analysis.

## CONCLUSION

MBT showed high specificity and negative predictive value, thus presenting an important value to rule out the occurrence of postoperative leaks.
